# Doppler Surrogate Endoscopy for Screening Esophageal Varices in Patients With Cirrhosis

**DOI:** 10.5812/hepatmon.11237

**Published:** 2014-01-16

**Authors:** Kambiz Akhavan Rezayat, Fariborz Mansour Ghanaei, Ahmad Alizadeh, Afshin Shafaghi, Ali Babaei Jandaghi

**Affiliations:** 1 Department of Internal Medicine, Imam Reza Hospital, School of Medicine, Mashhad University of Medical Sciences, Mashhad, IR Iran; 2 Division of Gastroenterology and Hepatology, Gastrointestinal and Liver Diseases Research Center (GLDRC), Guilan University of Medical Sciences, Rasht, IR Iran; 3 Department of Radiology, Poursina Hospital, Guilan University of Medical Sciences, Rasht, IR Iran

**Keywords:** Ultrasonography, Doppler, Duplex, Esophageal and Gastric Varices, Endoscopy

## Abstract

**Background:**

Portal hypertension is a common consequence of hepatic cirrhosis, which causes esophageal varices. Bleeding from varices has a high mortality rate. The present gold standard for diagnosing varices is endoscopy. Considering endoscopy side effects and patients' low acceptance, there have been always efforts for finding alternative diagnostic methods including Doppler ultrasonography (US).

**Objectives:**

The aim of the present study was to evaluate changes of Doppler indices in cirrhotic patients with and without esophageal varices.

**Patients and Methods:**

Sixty six patients with known cirrhosis entered this cross-sectional study. Gastroscopy was performed for patients, and the first questionnaire was filled based on the Japanese Portal Hypertension Society guidelines. Then patients were referred for Doppler US of splenoportal system, and information was documented in the second questionnaire.

**Results:**

Forty-four patients were male and 22 female. Forty six patients had esophageal varices, and 20 did not. There were no significant associations between splenoportal indices found by Doppler US, and presence of esophageal varices in patients. However, we found a negative association between platelet ratio to spleen diameter, and to splenic vein diameter.

**Conclusions:**

Neither of studied variables was perfect to differentiate cirrhotic patients with and without EVs. Endoscopy is still the gold standard diagnostic method for diagnosing esophageal varices in patients with cirrhosis. It seems that some of the splenoportal Doppler indices are promising, but more research and evaluation is necessary.

## 1. Background

Cirrhosis represents the end stage of progressive fibrosis which destroys normal liver tissue and produce regenerative nodules.

Variceal bleeding (which is a consequence of portal hypertension) is one of the most dreaded complications of cirrhosis. The risk of variceal bleeding is 25-40% in patients with cirrhosis (1, 2). When HPVG (hepatic venous pressure gradient) reaches 10 mmHg, the varices start to form, and when it reaches 12 mmHg the chance of bleeding increases dramatically (3). 

In this situation, to divert blood from portal system, collateral veins form in different places, which two important sites are distal esophagus and gastric; these sites contain most of bleeding from portal hypertension. The incidence of esophageal varices in patients without ascites is 40%, and 60% in those with ascites. The incidence of esophageal varices (EVs) increases almost 5% per year (4). Almost one third of patients with varices have eventually bleeding which is the main cause of mortality (5); the mortality rate of EVs bleeding reaches 15-20% (6-9). 

Survival of patients who had first variceal bleeding decreases dramatically, and only 30-40% of those who did not receive treatment survive two years after the initiation of bleeding (10).

Diagnosing gastric-esophageal varices and taking necessary precautions are important factors in reducing portal hypertension risks and increasing patients' survival. Endoscopy is the gold standard for diagnosing upper GI varices. Besides its diagnostic value, physicians can perform preventive or emergency treatment through endoscopy like sclerotherapy and band ligation (EVL). Based on the American Association for the Study of Liver Diseases (AASLD), all of patients with cirrhosis must undergo screening endoscopy to diagnose gastric-esophageal varices (11). Patients who do not have any sign of varices must be evaluated with endoscopy during the next three years. Patients who were diagnosed with varices at first endoscopy must undergo another endoscopy in the next two years (12). 

However, endoscopy even in its pure diagnostic form has high cost in most of the countries and has encountered by patients low acceptance. Therefore, many efforts have been performed to find a proper alternative method.

 Radiologic diagnostic methods even with double contrast do not have acceptable accuracy to diagnose varices in the early stages, and to determine hypertensive gastropathy changes appropriately. Therefore, they are not considered as good screening methods for varices, but in some studies, they showed high accuracy for diagnosing high-grade varices. One of the methods of interest for gastroenterologists and radiologists is Doppler US indices of splenoportal system, which shows extensive changes through cirrhosis and portal hypertension (13).

## 2. Objectives

The aim of the present study was to evaluate Doppler US indices of splenoportal system as a noninvasive, in hand, low cost and repeatable method for diagnosing esophageal varices in patients with cirrhosis.

## 3. Patients and Methods

This was a cross-sectional study with convenient sampling method, and the study population was all the patients with cirrhosis regardless of its etiology referred to us during 2011. Study was performed in Guilan Liver and GI Disease Research Center affiliated to Guilan University of Medical Sciences. Cirrhosis was diagnosed based on the clinical signs confirmed by laboratory and radiologic evaluations, and in rare cases was diagnosed with biopsy. Patients' age was not considered as an inclusion/exclusion criteria, but regarding sources of patient referral (patients who referred to GI Adult Clinic of Razi Hospital, Rasht), most of the patients were adults. The exclusion criteria were history of endoscopic treatment like sclerotherapy and band ligation, and history of medical treatment for reducing portal pressure. Patients with history of bleeding from varices (with or without endoscopic or medical intervention), patients with passive congestion of liver without cirrhosis, cirrhotic patients with known thrombosis in port or splenic vein (detected by Doppler), and patients with history of splenectomy were also excluded from the study. 

Sample size was calculated based on extracting the variance of portal vain indices measurement from the literature (14), measuring the effective clinical significance (d) by expert elicitation method, and considering the significant level (error of α) equal to 0.05.

Platelet count (the lowest documented amount) and upper GI endoscopy were performed for the patients. Endoscopic characteristics of portal system were documented based on the Japanese Portal Hypertension Society guidelines in four parts on the first questionnaire including; varices development in esophagus (upper, middle or lower third), color of varices (blue or white), presence or absence of red signs (including hematocystic spot-red, wale-cherry red, spot-diffuse redness), and grade (F1, F2, and F3). Then the patients were referred for Doppler US of splenoportal system, and findings (including portal, splenic and left gastric vein diameter, portal vein velocity, ultrasonic detectable gastroesophageal collaterals, flow direction in portal vein, respiratory variation of portal vein diameter, presence of ascites, splenic diameter, and splenoportal index) were documented in the second questionnaire. PENTAX EG 2940 with EPM 3500 processor made in Japan was used for endoscopy. For Doppler US, the Ultrasonix SP made in Canada with probe 3.5 was used. Endoscopy and Doppler US were all performed by a single experienced physician. 

The endoscopist and radiologist were unaware of the radiologic and endoscopic features, respectively. Since patients were in non-emergency condition, endoscopic and Doppler studies were all performed in the morning before the lunch time, which automatically eliminates the effects of probable differences in portal pressure in different times of day.

Using appropriate and advanced tools from known companies, performing endoscopy and Doppler US by experienced physicians, and preparing questionnaires based on accredited societies guidelines and indices from radiology and endoscopy reference sources, provided a proper validity to our gathered information.

SPSS version 16 was used to analyze the data, and t-test, Chi square and Spearmen correlation were employed to compare data. P value less than 0.05 was considered significant. 

## 4. Results

Sixty six patients entered the current study, including 44 male (66.7%). The mean age of patients was 55.39 ± 19.27 years. Forty six patients (69.7%) had esophageal varices, and 6 (9.1%) had gastric varices. Gender and the mean age of patients did not show significant difference between those patients with and without EVs (P = 0.449 and P > 0.05, respectively). T-test did not determine any significant difference between those patients with and without esophageal varices for portal vein diameter, splenic vein diameter, left gastric vein diameter, the mean platelet count, and the blood velocity in portal vein (P > 0.05 for all). By using Chi Square, we also could not find any significant difference between those patients with and without EVs for portal vein diameter changes during respiration, blood flow direction in portal vein, ultrasonic detectable gastro-esophageal collaterals, presence of gastric varices, presence of ascites and portal vein velocity (P = 0.503, P = 0.503, P = 0.785, P = 0.09, P = 0.254, P = 0.424, respectively). Splenoportal index (SPI), spleen diameter, and platelet spleen diameter ratio did not have significant difference between the two groups with and without EVs (P = 0.165, P = 0.175, P = 0.495, respectively). Spearman Rho test was used to find correlation between platelet count and varices grading, and between platelet count and spleen diameter ratio and varices grading (P = 0.938, P = 0.710, respectively). Also we found a negative 

correlation between platelet counts spleen diameter ratio, also splenic vein diameter. However, no significant difference was observed between patients with and without esophageal varices by each of these parameters separately. Some of the patients` demographic data and diagnostic findings are shown in Table 1. 

**Table 1.** Patients Demographic Data and Diagnostic Findings

Variable	With Esophageal Varices	Without Esophageal Varices	Total
**Gender Number, %**			
Male	32 (69.6)	12 (60)	44 (66.7)
Female	14 (30.4)	8 (40)	22 (33.3)
**Age, Patient, y, Number (mean)**	45 (53.91)	20 (59)	P = 0.333
**Portal vein diameter, mm, Number (mean)**	46 (12.7)	19 (12.79)	P = 0.912
**Splenic vein diameter, mm, Number (mean)**	44 (10.8)	19 (9.4)	P = 0.191
**Left coronary vein diameter, mm, Number (mean)**	41 (4.71)	19 (4.05)	P = 0.356
**Platelet, /µL, Number (mean)**	46 (99826)	20 (102450)	P = 0.885
**Respiratory Changes of Portal Vein, No. (%)**			
> 50	1 (2.3)	0 (0)	1 (1.6)
< 50	42 (97.7)	19 (100)	61 (98.4)
**Blood flow speed in port vein, cm/sec, Number (mean)**	43 (16.86)	19 (18.28)	P = 0.424
**Gastro-Esophageal Collateral, No. (%)**			
Present	13 (28.3)	5 (25)	18 (27.3)
Not present	33 (71.7)	15 (75)	48 (72.7)
**SPI, Number (mean)**	42 (5.82)	19 (4.61)	P = 0.165
**Mean platelet /spleen Diameter, Number (mean)**	45 (676.65)	20 (773.11)	P = 0.495
**Spleen diameter, mm, Number (mean)**	45 (160.11)	20 (147.1)	P = 0.175
**Gastric Varices, No (%)**			
Present	6 (13)	0 (0)	6 (9.1)
Not present	40 (87)	20 (100)	60 (90.9)
**Ascites, No. (%)**			
Present	32 (69.6)	11 (55)	43 (65.2)
Not present	14 (30.4)	9 (45)	23 (34.8)

## 5. Discussion

Esophageal varices bleeding is accompanied with poor prognosis and decreases patient survival. Increase of portal vein diameter more than 13 mm represents portal hypertension with a specificity of 95-100% and a sensitivity of 42% (15, 16). In our study, the mean of portal vein diameter in patients without EVs was 12.79, and 12.7 in those with varices, which did not have significant difference, and both were lower than the cutoff point (13 mm). This finding acknowledged the fact that with progression of portal hypertension, new collaterals appear, and diameter of present collaterals increase, so the portal blood flow diverts and portal vein diameter decreases (14). 

In a half of patients with portal hypertension, the splenic vein diameter increases to more than 10 mm (15). The mean diameter of splenic vein in our patients without EVs was 9.4 and 10.8 in those with EVs, but the difference was not significant. The reasons could be first the splenic vein diameter changes only in 50% of patients, and the second one of the main parameters for splenic vein hypertension is gastric varices, especially in fundus; in our study only six patients had gastric varices. It seems that in our patients most of collaterals had formed in other sites, so we did not observe statistically significant difference between the two groups. 

The mean diameter of the left gastric vein in our patients without EVs was 4.05 mm, and was 4.71 in those with EVs , but the difference was not significant. In one study increasing left coronary vein diameter of more than 5 mm (17), and in another more than 6 mm (18) were considered as a sign of portal hypertension with 80% sensitivity. This dissimilarity between our results and reported results may be due to the fact that evaluation of left gastric vein is not very easy in Doppler US, because of its small diameter and place, as we could not find it in 7 of our patients, and the fact that the left coronary vein is in direct relation with port, and when collaterals are sufficient to reduced portal dimension like our patients, the pressure decreases in the left coronary vein as well.

In healthy persons, the diameter of splenic and portal veins increase 50-100% during respiration, in patients with portal hypertension these reduce to less than 50% (15, 16). In our study most of the patients had respiratory changes less than 50%, and the difference between patients with and without EVs was not significant. It seems that these changes happened in early stages of portal hypertension even before gastric-esophageal varices development, therefore cannot differentiate between the two groups of patients (with and without EVs).

Splenic index is spleen length multiply by its width (cm), by dividing splenic index on blood velocity in port vein the splenoportal index (SPI) is calculated. In the present study, SPI was 4.61 in patients without EVs, and 5.28 in those with it. Using the Roc method, the cutoff point of SPI was 4.15 with sensitivity and specificity of 33.3% and 31.5%, respectfully. The low sensitivity and specificity of this index make it invaluable for diagnosing esophageal varices. 

With progressing of hepatic fibrosis and portal hypertension, the splenic vein diameter increases and consequently platelet number decreases, therefore platelet count/spleen diameter ratio is an index for evaluating varices. Giannini from Italy considered cutoff point of 909 as a reliable index for diagnosing esophageal varices (20). Other studies also reported a cutoff point between 160-1014 (20-22), however, Saewar in his recent study did not find any significant association between platelet count/ spleen diameter ratio and EVs (23).

In the present study, the mentioned ratio was 773.11 in patients without EVs, and 676.65 in those with EVs, but the difference was not significant (P = 0.495). Based on the Roc cure diagram the cutoff point of 759.01 had the highest sensitivity and specificity of 68.8% and 25%, respectively, the low sensitivity and specificity of this index, make it unsuitable to differentiate patients with and without EVs. 

In this study we also evaluated some other indices including spleen diameter, frequency of gastric varices, presence of ascites, and two variable indices including splenic vein diameter and blood velocity, portal vein diameter and blood velocity, left gastric vein and blood velocity, blood velocity to spleen diameter, and blood velocity to SPI. 

Although, we did not find any statistically significant difference between patients with and without EVs for these variables, however the blood velocity in portal vein to splenic vein diameter (P = 0.056) and blood velocity in portal vein to spleen diameter (P = 0.07) worth further evaluation in other studies with larger sample size. 

Liu et al. conducted a study on 383 cirrhotic patients with Child score A for diagnosing EVs with Doppler US. His results indicated that cutoff value of 3 for SPI have a sensitivity of 92%, specificity of 93%, positive predictive value (PPV) of 91%, and Negative predictive value (NPV) of 94% for diagnosing EVs. He concluded that this cut off had capability of diagnosing EVs in 92% of patients who did not have endoscopy, and therefore is a reliable index (19). 

Dib et al. from France stated that although using noninvasive method for diagnosing EVs is logical and rational, but still endoscopy is the preferable and the most reliable method compared with other diagnostic methods, however we have to expect more studies on capsule endoscopy (24). 

### 5.1. Limitations 

The limitations of this study were low number of cases and controls, variability of the velocity, and differences in diameter and pressure of the veins in different times of day. 

In the present study, neither of studied variables was perfect to differentiate between cirrhotic patients with and without EVs. Further studies with larger sample size may indicate value of two variable indices; blood velocity to splenic vein diameter, and blood velocity to spleen diameter. Endoscopy is still the gold standard and accurate diagnostic method to diagnose gastric-esophageal varices.
